# The Prevalence of Irritable Bowel Syndrome and Its Relation to Psychiatric Disorders Among Citizens of Makkah Region, Saudi Arabia

**DOI:** 10.7759/cureus.32705

**Published:** 2022-12-19

**Authors:** Malik H Alharbi, Ahmad H Alhazmi, Mohammad H Ujaimi, Moath Alsarei, Mansour M Alafifi, Fawaz S Baalaraj, Mokhtar Shatla

**Affiliations:** 1 Department of Medicine, Faculty of Medicine, Umm Al Qura University, Makkah, SAU; 2 Department of Family Medicine, College of Medicine, Umm Al Qura University, Makkah, SAU

**Keywords:** saudi arabia, rome iv criteria, anxiety, depression, phycological risk factor, irritable bowel syndrome

## Abstract

Background and aim: Irritable bowel syndrome (IBS) is a chronic functional bowel disorder. Many adults worldwide have symptoms associated with IBS and are responsible for most gastroenterology visits. The aim of this study is to illustrate and analyze the prevalence of IBS among the general population in Makkah Al-Mukarramah city using the Rome IV criteria in relation to psychiatric disorders.

Methodology: This was a cross-sectional study conducted on Makkah citizens. The study excluded all residents in Makkah without Saudi nationality or below the age of 18. The survey was created using Google forms and shared randomly on social media. The sample size was calculated using the OpenEpi website v3.0. The online questionnaire is composed of three sections: sociodemographic data, Rome IV criteria with the Bristol Stool Scale, and lastly the Depression, Anxiety, and Stress Scale 21 (DASS-21) score for stress, anxiety, and depression. Data were analyzed using SPSS software for Windows V.23, and odds ratio (OR) with 95% confidence intervals (95% CI) was obtained for selected risk factors using logistic regression.

Results: Nine hundred and twenty-one individuals from Makkah city completed the survey. The overall IBS prevalence was 20.19%. The commonest subtypes of IBS were IBS-M followed by IBS-C (53.8% and 22%, respectively). In the multiple regression analysis, stress (P = <0.001, OR = 2.473) was statistically significantly associated with IBS.

Conclusion: In this study, the prevalence of IBS among Makkah citizens is high. Stress was found to be a major risk factor for IBS.

## Introduction

Irritable bowel syndrome (IBS) is a chronic condition that is classified as a functional bowel disorder. The main complaint of patients with IBS can be abdominal pain and discomfort or changes in bowel habits [1]. Approximately, 10%-20% of adults worldwide suffer from symptoms that can be associated with IBS, with women and adults who are less than 50 years old being more vulnerable to developing IBS [2]. IBS is a common functional bowel disorder being the most recognized cause of gastroenterologist visits and one of the most commonly diagnosed gastrointestinal conditions [3]. It can affect a wide scope of ages and economic, social, and ethnic groups. This results in an economic burden on the healthcare system and can severely impair the quality of life of the patients. The exact etiology of IBS is poorly understood [3,4]. IBS diagnosis can be difficult due to the absence of objective diagnostic findings, making it restricted merely to medical history. Currently, the diagnosis of IBS is made based on the Rome IV criteria, which is consist of recurrent abdominal pain that occurred one day per week in the last three months with the association of pain related to defecation, changes in stool frequency, and appearance [5,6]. During the process of diagnosing patients with IBS, they might face many difficulties such as stigmatization or discriminatory action. Which can delay the treatment plan further and might take up to four years to make an accurate diagnosis [7]. IBS symptoms can be triggered by several risk factors, including chronic stress [8], anxiety, depression [9], and smoking [10]. A cross-sectional study conducted in a tertiary medical center in Jeddah discovered a strong correlation with fibromyalgia, headache, and migraines [11]. According to the Rome IV criteria, a recent study conducted in Jazan found that the overall prevalence of IBS among the Jazan general population in Saudi Arabia was 16%. Females had a significantly higher incidence than males. Also, stress, anxiety and tobacco smoking were statistically significant [12]. Another study conducted on medical students and interns at King Abdulaziz University in Jeddah used a self-administered questionnaire based on Rome III criteria to diagnose IBS. The study showed a prevalence of IBS at 31.8% and showed a correlation between IBS and female gender, anxiety, emotional stress, living in a school dormitory away from family, fifth and sixth-year medical students, and interns [13]. A study conducted on the general population in central Saudi Arabia revealed an overall IBS prevalence of 30.5% using Rome III criteria [14]. In another study conducted on a total of 767 Saudi undergraduates aged 18 or older across all regions in Saudi Arabia using the Rome IV criteria questionnaire, 15.8% of the participants met the Rome IV criteria for IBS diagnosis with higher rates among females, medical students, tobacco smokers, positive family history of IBS in the first-degree relatives, emotional stress, and poor lifestyle [15]. Also, a cross-sectional study that included 1,680 participants from different regions of Saudi Arabia revealed that the prevalence of IBS was 18.2%. IBS-M was the most common subtype among IBS patients (42.3%). One study found that risk factors that are significantly associated with IBS were smoking, gastroesophageal reflux disease (GERD), food allergy, anxiety, psychological stress, family history of IBS, regular use of non-steroidal anti-inflammatory drugs (NSAIDs) history of infection before the occurrence of symptoms and residence in the south of Saudi Arabia [16] A recent cross-sectional study was conducted in 2021 included 230 students in female secondary schools in Arar city revealed the overall prevalence of IBS among the studied group is 54.8% [17]. Another study that included 401 students in female secondary schools revealed the overall prevalence of IBS was 21.4% in Ar Rass city, Qassim region, Saudi Arabia [18]. Although numerous studies conducted in many regions in Saudi Arabia, there was a lack of studies conducted specifically on Makkah Al-Mukarramah city to measure the prevalence and the associated risk factors. Therefore, our aim is to measure the prevalence of IBS among the general population in Makkah Al-Mukarramah city in relation to psychiatric disorders.

## Materials and methods

This is a descriptive cross-sectional study conducted by an online survey using (Google Forms) and shared survey among Makkah citizens from February to April 2022. The study was done exclusively in Makkah city, Saudi Arabia. The survey was shared freely on social media, such as WhatsApp, between Makkah citizens.

The survey was collected from different categories, different gender, and different ages through convenience sampling. The required sample size for this study was calculated by the OpenEpi website version 3.0 [19]. The population size of Makkah city is about 1,675,368 inhabitants [20], keeping the confidence interval (CI) level at 95% and considering a 50% prevalence of knowledge about IBS. Therefore, the sample size required to achieve it is 385 participants. We include Makkah citizens and exclude Makkah residents and participants below the age of 18.

The questionnaire included three sections. The first section includes demographic information. The validated Rome IV questionnaire for IBS in adults was used in the second section. IBS is one of the numerous functional GI diseases evaluated by the Rome IV questionnaire [21]. Based on the Bristol Stool Scale, the Rome IV questionnaire also divides IBS patients into the following four subtypes: constipation-predominant (IBS-C), diarrheal (IBS-D), mixed (IBS-M), or unclassified (IBS-U). The Rome Foundation provided a licensing agreement for the use of the Rome IV diagnostic questionnaire with an Arabic translation. The final section of the questionnaire asked about stress, anxiety, and depression. Questions on stress, anxiety, and depression were taken from the Depression, Anxiety, and Stress Scale 21 (DASS-21) Arabic-validated version. The DASS-21 rates participant perceptions on a 4-point Likert scale. Cut-off scores for these disorders were 8 or more for anxiety, 15 or more for stress, and 10 or more for depression [22]. Data are collected via an excel sheet and then the analysis was done in SPSS software for Windows, version 23. Frequencies and percentages were used for the descriptive analysis, and a chi-square test was done to find the relation between categorical variables. Variables that showed significant association in the bivariate analysis were included in an enter method multiple logistic regression analysis. P-value < 0.05 was considered statistically significant.

## Results

A total of 921 participants completed the questionnaire. Table 1 presents the sociodemographic data of the study. Most of the study group were aged between 18-30 years old (80.5%). Most of whom completed the questionnaire were females, 547 (59.4%) compared to 374 (40.6%) males. 72.6% of the study group were singles compared with 27.3% who were married, divorced, or widower. Most of the participants were non-smokers 761 (82.6%), smokers 121 (13.1%), and previous smokers 39 (4.2%). The majority of the participants were students 583 (63.3%), employers were 188 (20.5%) and 150 (16.3%) were retired or unemployed. 72% of the participants' income was less than 5000 riyals. The majority of the participants have a university degree or higher (80%). 422 (45.9%) participants have normal body mass index (BMI), 205 (22.3%) were overweight, 150 (16.3%) were obese and the least were underweight 142 (15.5%).

**Table 1:** Sociodemographic data according to irritable bowel syndrome prevalence

Variables	N (Total=921)	%
Age:		
18-30	741	80.5
31-40	71	7.7
41-50	54	5.9
51-60	43	4.7
>60	12	1.3
Gender		
Male	374	40.6
Female	547	59.4
Social status:		
Single	669	72.6
Married	227	24.6
Divorced	20	2.2
Widower	5	0.5
Smoking status:		
Smoker	121	13.1
Non-smoker	761	82.6
Previous smoker	39	4.2
Employment status:		
Student	583	63.3
Self-employment	23	2.5
Employee in the government sector	112	12.2
Employee in the private sector	53	5.8
Retired	35	3.8
Not working	115	12.5
Income		
Less than 5000 riyals	663	72.0
5000-10000 riyals	123	13.4
>10000 riyals	135	14.7
Education:		
High school or below	184	20.0
University or higher	737	80.0
BMI:		
Underweight (< 18.5 kg/m^2^)	142	15.4
Normal (18.5-24.9 kg/m^2^)	424	46.0
Overweight (25-29.9 kg/m^2^)	205	22.3
Obese (>30 kg/m^2^)	150	16.3

Participants above 60 years old showed the highest prevalence of IBS (25.0%), female gender (21.0%) were slightly higher than male gender (19.0%). However, the difference is not significant. Social status among participants showed no significance in the prevalence of IBS. Previous smokers (23.1%) were more likely to have IBS than smokers (22.3%) and non-smokers (19.7%). However, it is not significant. Employment status, income, education level and BMI have no statistical significance in prevalence of IBS. Depression, anxiety and stress showed a statistically significant with a P-value < 0.001.

**Table 2:** Relation between irritable bowel syndrome and each of the sociodemographic data, depression, anxiety, and stress

Irritable bowel syndrome association with selected risk factors				
Variables		Yes (%)	No (%)	P-value
Age	18-30	153 (20.6)	588 (79.4)	0.432
	31-40	17 (23.9)	54 (76.1)	
	41-50	8 (14.8)	46 (85.2)	
	51-60	5 (11.6)	38 (88.4)	
	>60	3 (25.0)	9 (75.0)	
Gender	Male	71 (19.0)	303 (81.0)	0.449
	Female	115 (21.0)	432 (79.0)	
Social status	Single	142 (21.2)	527 (78.8)	0.342
	Married	38 (16.7)	189 (83.3)	
	Divorced	4 (20.0)	16 (80.0)	
	Widower	2 (40.0)	3 (60.0)	
Smoking status	Smoker	27 (22.3)	94 (77.7)	0.723
	Non-smoker	150 (19.7)	611 (80.3)	
	Previous smoker	9 (23.1)	30 (76.9)	
Employment status	Student	120 (20.6)	463 (79.4)	0.374
	Self-employment	6 (26.1)	17 (73.9)	
	Employee in the government sector	28 (25.0)	84 (75.0)	
	Employee in the private sector	10 (18.9)	43 (81.1)	
	Retired	4 (11.4)	31 (88.6)	
	Not working	6 (26.1)	97 (84.3)	
Income	Less than 5000 riyals	125 (18.9)	538 (81.1)	0.216
	5000-10000 riyals	27 (22.0)	96 (78.0)	
	>10000 riyals	34 (25.2)	101 (74.8)	
Education	High school or below	35 (19.0)	149 (81.0)	0.658
	University or higher	151 (20.5)	586 (79.5)	
BMI	Underweight	33 (23.2)	109 (76.8)	0.307
	Normal	74 (17.5)	350 (82.5)	
	Overweight	46 (22.4)	159 (77.6)	
	Obese	33 (22.0)	117 (78.0)	
Depression	Yes	147 (25.6)	427 (74.4)	<0.001
	No	39 (11.2)	308 (88.8)	
Anxiety	Yes	150 (25.5)	438 (74.5)	<0.001
	No	36 (10.8)	297 (89.2)	
Stress	Yes	141 (28.7)	351 (71.3)	<0.001
	No	45 (10.5)	384 (89.5)	

Table 3 describes the prevalence of the IBS and its subtypes in Makkah population, the prevalence of IBS is (20.2%), then divided into IBS-M (mixed subtype) 53.8% followed by IBS-C (constipation subtype) by 22%, followed by IBS-D (diarrhea subtype) (15%) then IBS-U (unsubtyped) (8.6%).

**Table 3:** Irritable bowel syndrome subtypes

Irritable bowel syndrome subtypes	N (%)
IBS-Constipation subtype	41 (22.0)
IBS-Diarrhea subtype	29 (15.6)
IBS-Mixed subtype	100 (53.8)
IBS-Unspecified	16 (8.6)

Table 4 describes the association between IBS and depression, anxiety and stress disorder patients. The risk of developing IBS while having stress disorder was statistically significant with P-value of <0.001 and odd ratio (OR) of 2.473. While the risk of developing IBS when having anxiety or depression were statistically insignificant; P-value of 0.149 for anxiety and P-value of 0.367 for depression.

**Table 4:** Multiple logistic regression analysis for selected irritable bowel syndrome risk factors

Variables	β (SE)	P-value	OR	95% CI (OR)	
				Lower	Upper
Depression (Yes)	0.232 (0.258)	0.367	1.261	0.761	2.089
Anxiety (Yes)	0.368 (0.255)	0.149	1.445	0.876	2.384
Stress (Yes)	0.905 (0.242)	<0.001	2.473	1.539	3.973

## Discussion

The aim of this study was to explore the prevalence and risk factors of IBS in Saudi Arabia in Makkah. Our results demonstrate that the prevalence of IBS was 20.19% (applying Rome IV criteria), it thus falls within the IBS prevalence range globally [2]. However, it is higher than the findings of a recent systematic review and meta-analysis from 34 countries, which included 82,476 participants with a prevalence of 3.8% using the Rome IV criteria [23]. In middle eastern countries, there is inadequate statistical data about IBS. However, the prevalence of IBS can be strongly influenced by cultural differences, various procedures for data collection, and many other factors [12]. For instance, Egypt has the highest prevalence rate of IBS among all nations at 7.6%, according to Rome Foundation research that covered 33 countries, where it was the lowest in Singapore with 1.3% [24]. Furthermore, another study conducted on Lebanon's general population revealed a high prevalence of 20.1% using the Rome III criteria [25]. Our research revealed that the prevalence of IBS was comparable to that reported in Saudi Arabia, which is between 16% to 31.8% [12-14,16]. According to epidemiological data from a recent IBS study, IBS-M (mixed subtype) seems to be the most common subtype [26]. The findings of our study indicate that IBS-M (mixed subtype) was the most common subtype of IBS among participants, accounting for 53.8% of cases, followed by IBS-C (constipation subtype), accounting for 22% of cases; this systematic pattern of the two subtypes is consistent with many other studies conducted in Saudi Arabia [16,27,28].

In our study, female participants were more likely to be affected by IBS than men. However, there was no statistical significance between the male and female gender. However, contrary to a study done in Saudi Arabia, showed that the female gender has a significant correlation with IBS [12], and several other studies were conducted in western countries [15,29,30]. Age and socioeconomic status, on the other hand, failed to demonstrate statistical significance, which is a result that is consistent with a global meta-analysis [31]. Furthermore, our study exhibited results similar to numerous previous studies with not statistically significant for social [13], employment [16], and educational status [32]. Our study did not show statistical significance in the association between IBS and smoking and BMI. Likewise, to the previous study done in 2015 [33,34]. Regarding psychological risk factors, participants with anxiety and depression in the present study had a statistically significant higher likelihood to have IBS than those without them, where patients diagnosed with stress were 2.5 times more likely to have IBS than an intact person, which points to brain-gut interaction and problems arise when mood disorders disrupt hemostasis, altering gut motility and visceral sensitivity as well as releasing neuropeptide and microbiota, which ultimately leads to the development of IBS [35,36]. The result of our study is consistent with three other studies, one done in China [37] and another done in Riyadh among medical students [38], and the last one done in the general population of Jazan, Saudi Arabia [12]. However, one study found a correlation between IBS and anxiety only with no correlation with depression.

Although the present study provided information about the prevalence of IBS and its associated risk factors in the Makkah population, it still has some limitations. The first limitation is the questionnaire was filled out through online self-administration and was not interviewed directly; This may reduce the reliability of participants' answers. Additionally, participants who met the IBS diagnostic criteria in our cross-sectional study were not sufficiently investigated to rule out other possible diagnoses. Moreover, the cross-sectional methodology used in this study could not establish causation between IBS and the risk factors. On the other hand, the strength of this study includes using validated and Arabic-translated Rome IV scale and DASS-21 questionnaire.

## Conclusions

This study contributes to understanding the prevalence of IBS and the associated risk factors. It demonstrated the prevalence of IBS among the Makkah population, using the ROME IV scale. The study showed a high prevalence of IBS in Makkah city, and it is associated significantly with stress, which is consistent with other studies in the region.
